# Effect of Combination of Chinese Herbal Medicine versus Western Medicine on Mortality in Patients after Cardiopulmonary Resuscitation: A Systematic Review and Meta-Analysis of Randomized Controlled Trials

**DOI:** 10.1155/2016/3506757

**Published:** 2016-02-03

**Authors:** Wenxiu Guo, Xiaoguang Lu, Dalong Wang, Tuo Chen, Zhiwei Fan, Yi Song

**Affiliations:** ^1^Dalian Medical University, Dalian 116044, China; ^2^Department of Emergency Medicine, Affiliated Zhongshan Hospital of Dalian University, Dalian 116001, China

## Abstract

*Introduction*. Although Chinese herbal medicine (CHM) treatment combined with conventional western therapy has been widely used and reported in many clinical trials in China, there is uncertainty about the efficacy of this combination in the treatment of patients after cardiopulmonary resuscitation (CPR). This systematic review aimed to assess whether the risk of mortality has decreased comparing the combination of CHM treatment with conventional western therapy.* Methods*. To identify relevant studies, the literature search was conducted in Medline, Embase, the Cochrane Library, CBM, CNKI, VIP, and Wanfang database. We included all randomized controlled trials (RCTs) that compared outcomes of patients after CPR taking combination of CHM treatment with those taking just conventional western therapy.* Results*. This meta-analysis showed that patients randomly assigned to combined CHM treatment group had a statistically significant 23% reduction in mortality compared with those randomly assigned to conventional western therapy group (RR: 0.77; 95% CI: 0.70–0.84).* Conclusions*. This meta-analysis provides evidence suggesting that a combined CHM therapy is associated with a decreased risk of mortality compared with conventional western therapy in patients after CPR. Further studies are needed to provide more evidence to prove or refute our conclusion and identify reasons for the reduction of mortality.

## 1. Introduction

Significant progress has been made during the past half century on improving rate of return of spontaneous circulation (ROSC) by means of modern cardiopulmonary resuscitation (CPR). Nonetheless, mortality of resuscitated patients remains unacceptably high, with population-based studies continuing to report rates of 61%–90% [1–3].

Reasons for the high mortality rate of patients who initially achieve ROSC after experiencing cardiac arrest can be attributed to a unique and complex combination of pathophysiological processes involving multiple organs [4]. Although ischemia of the whole body initially causes global tissue and organ injury, the critical additional damage on multiple organ systems occurs during and after reperfusion [5, 6]. The features of pathophysiology after ROSC are often superimposed by the disease or injury that caused the cardiac arrest as well as underlying complications, especially systemic inflammatory response syndrome (SIRS) and multiple organ failure (MODS). Therapies that act on individual organs may aggravate other injured organ systems. The severity of the disease is also associated with the cause of cardiac arrest, the severity of the ischemic insult, and the patient's physical condition which will vary with these factors.

At present, the main conventional western treatments include intensive care unit (ICU) management, early hemodynamic optimization, oxygenation, ventilation, circulatory support, management of acute coronary syndrome (ACS), and other persistent precipitating pathologies. Although there are many treatments, there is still lack of substantial progress in decreasing the mortality for patients after CPR. Therefore, an adjunctive or a combined approach should be found to solve this urgent problem.

In traditional Chinese medicine (TCM), the pathophysiological status of patients after CPR was first seen in the description of the Chinese classic medical book* Inner Classic of the Yellow Emperor* (475 BC–221 BC). It belongs to the category of “Jue-Tuo syndrome” with the main clinical characteristics of pale/dim complexion, cyanosis, cold limbs, profuse perspiration, haziness or dysphoria, weak breathing and pulse, even hyperpyrexia and coma, and so forth. In recent years, CHM treatment combined with conventional western therapy (combined CHM therapy) has been widely used to treat patients after CPR. A large number of clinical trials [7–14] have proved that the approach of combined CHM therapy could decrease mortality and improve prognosis. This systematic review aims to summarize the current research results on the effectiveness of combined CHM therapy on the risk of mortality and the complication rate in patients after CPR.

## 2. Materials and Methods

### 2.1. Search Strategy and Selection Criteria

A systematic review was conducted in accordance with PRISMA (Preferred Reporting Items for Systematic Reviews and Meta-Analyses) guidelines [15]. The research question, search strategy, inclusion criteria, and statistical analyses were prespecified. The literature search, data extraction, and quality assessment were done independently by two reviewers (DL Wang and WX Guo). Relevant studies were identified by systematic searches in Medline, Embase, the Cochrane Library, CBM, CNKI, VIP, and Wanfang databases from the date of inception until May, 2015. The searching terms were “Cardiac Arrest”, “Heart Arrest”, “Cardiopulmonary Resuscitation”, “advanced cardiac life support”, “post-resuscitation or post-cardiac arrest/post-cardiac arrest syndrome” and “traditional Chinese medicine”, “integrative medicine”, “alternative-medicine”, and “Chinese herbal medicine”. Additionally, reference lists of identified trials and review articles were screened for further relevant publications. There were no restrictions for language, publication date, or publication status.

All completed RCTs that assessed the effects of combined CHM therapy versus conventional western therapy in adult patients after ROSC were eligible for inclusion.

In addition, studies had to investigate all-cause mortality since it was the primary outcome. The secondary outcome was the complication rate. Duplicates were identified and deleted. Reviews, animal studies, case reports, editorials, letters, comments, and conference abstracts were excluded.

### 2.2. Data Extraction

The obtained articles were evaluated by two reviewers (WX Guo and DL Wang) in accordance with a preconfigured form. For each study, the following variables were extracted: study design, authors, date of publication, patients' characteristics including age, gender, and quantity, Chinese herbal treatment protocol, control intervention, and outcome parameters, length/time of follow-up. Disagreements between reviewers were resolved by a third reviewer (Xiaoguang Lu).

### 2.3. Quality Assessment

The risk of bias was analyzed using the assessment tool from the Cochrane Handbook [16], which included seven domains: random sequence generation, allocation concealment, blinding of participants and personnel, blinding of outcome assessment, incomplete outcome data, selective reporting, and other sources of bias. “L,” “U,” and “H” were used as a key for the judgement of each domain, with “low” (L) indicating a low risk of bias, “unclear” (U) indicating that the risk of bias was unclear, and “high” (H) indicating a high risk of bias.

### 2.4. Data Synthesis and Analysis

We used Review Manager (RM 5.2), provided by the Cochrane Collaborations for data analysis. For dichotomous data, risk ratio (RR) with 95% confidence interval (CI) was used to present the treatment effect. For continuous data, we presented the treatment effect as the mean difference (MD) with 95% CI. Fixed-effects models and/or random-effects models were used to calculate MD and/or RR based on the heterogeneity. Heterogeneity was identified by visual inspection of the forest plots and assessed by using a standard Chi-squared test with a significance level of *P* < 0.1. At the same time, heterogeneity was examined with *I* ^2^, where *I* ^2^ values over 50% indicate a substantial level of heterogeneity [16] and diversity [17]. We used the funnel plot to evaluate the potential publication bias.

## 3. Results

### 3.1. Study Search and Selection

**Figure 1:**
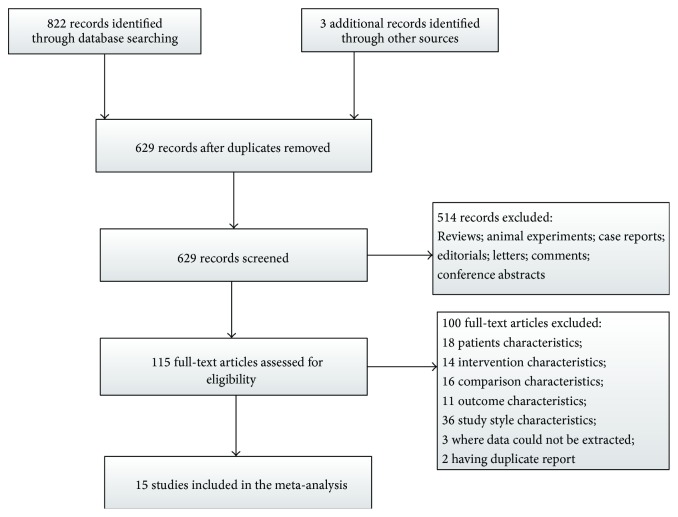
Flow chart of study selection.

We yielded a total of 825 publications through electronic searches. After 196 duplicates discarded, we excluded publications based on titles and/or abstracts, mainly because they are reviews, animal studies, case reports, editorials, letters, comments, and conference abstracts. 115 full-text studies were identified for further assessment. Subsequently, studies were excluded in line with the principle of PICOS (Patients-Intervention-Comparison-Outcome-Study Style). Of these, we excluded 100 studies which are mainly irrelevant to the PICOS: the characteristics of patients (18 studies), intervention (14 studies), comparison (16 studies), outcome (11 studies), study style (36 studies), studies where data could not be extracted (3 studies), and duplicate reports (2 studies). Accordingly, 15 studies fulfilled the inclusion criteria and were included in this systematic review. For a summary of the study selection process see Figure 1.

### 3.2. Description of the Studies

Table 1 summarizes the characteristics of the 15 included trials which were all carried out in China and published in Chinese journals. All trials were RCTs. There were a total of 1066 participants over all included studies. Sample sizes varied from 40 to 250, with an average of 49.82 participants per trial. With the exception of two studies [18, 19], all other included studies reported the average age of the participants. The year of publication ranged from 2004 to 2014. 12 trials reported combined CHM therapy versus conventional western therapy, 2 trials [20, 21] reported combined CHM therapy versus conventional western therapy plus another western medicine, and 1 trial [19] reported combined CHM therapy versus conventional western therapy plus placebo. For the application of CHM in the combined CHM therapy group, 8 studies [19, 20, 22–27] reported the use of Shenfu injection (extracted from* Renshen and Fuzi*), 2 studies [21, 28] reported the use of Shengmai injection (extracted from* Renshen*,* Maidong*,* and Wuweizi*), 2 studies [29, 30] reported the use of Xuebijing injection (extracted from* Honghua*,* Chishao*,* Chuanxiong*,* Danshen*,* and Danggui*), 1 study [18] reported the use of Erhuang powder (composed of* Daihuang and Huangqi*), 1 study [31] reported the use of Sini decoction (composed of* Ganjiang*,* Fuzi*,* and Zhigancao*), and 1 study [32] reported the use of Xuefuzhuyujiawei decoction (composed of* Renshen*,* Fuzi*,* Honghua*,* Chishao*,* Chuanxiong*,* Danggui*,* Huangqi*,* Taoren*,* Shengdihuang*,* Niuxi*,* Jiegeng*,* Chaihu*,* Zhiqiao*,* and Gancao*). There was one multicentre trial [22]. The duration of the treatment ranged from 3 days to 14 days. With regard to the outcome measures, all trials [18–32] reported the mortality. Seven studies [18, 20–22, 27–29] reported the complication rate; among them 5 studies [20–22, 27, 28] reported the incidence of arrhythmia, 1 study [18] reported the incidence of stress-induced gastrointestinal mucosal lesions and toxic enteroparalysis, and 1 study [29] reported incidence of postresuscitation syndrome (PRS).

### 3.3. Methodological Quality of Included Studies

**Figure 2:**
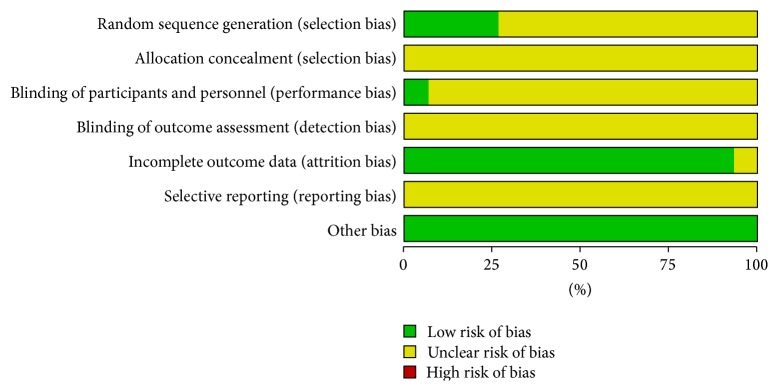
Methodological quality graph: review authors' judgements about each methodological quality item presented as percentages across all included studies.

**Figure 3:**
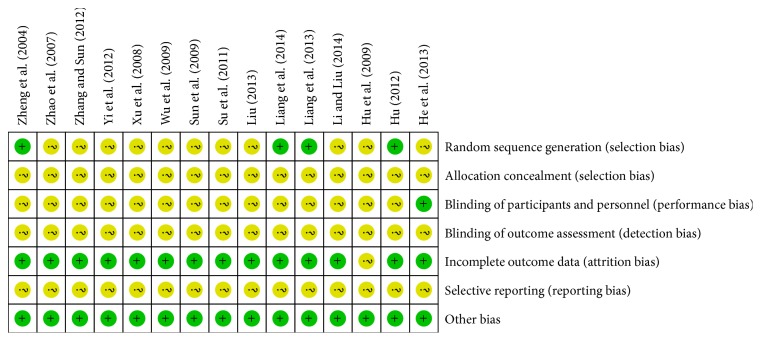
Methodological quality summary: review authors' judgements about each methodological quality item for each included study. +: L (low risk of bias); ?: U (unclear risk of bias); −: H (high risk of bias).

The risk of bias of included trials was summarized in Figures 2 and 3. According to the Cochrane Collaboration criteria, all of the included trials were evaluated as unclear methodological quality. 15 included trials were described as RCTs. Only 4 trials [24, 25, 30, 31] described the random method for generating random sequences. There was no description on the method of allocation concealment in any of the trials. Only one study mentioned blinding and placebo-controlled method [19]. The number of people at follow-up and the reasons for the failures at follow-up were not reported in any of the trials. There were no withdrawals or dropouts in any of our included trials and all trials reported complete clinical outcome data. The characteristics of participants in different treatment groups of studies were similar and comparable at baseline (gender, age, disease severity, etc.).

### 3.4. Effects of Interventions

#### 3.4.1. The All-Cause Mortality

**Figure 4:**
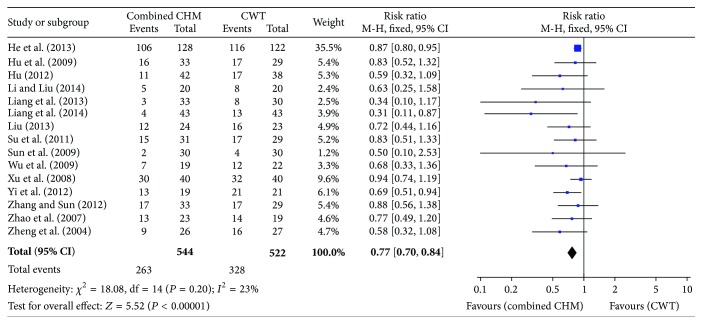
Forest plot of all-cause mortality.

15 studies reported the all-cause mortality, with a total sample of 1066 patients (544 in the combined CHM therapy group and 522 in the CWT group) [18–31]. A fixed-effects model was used to analyze the data according to the minimal heterogeneity (*P* = 0.20; *I* ^2^ = 23%). The meta-analysis of these studies showed a significantly decreased risk of mortality for patients after CPR treated with combined CHM therapy compared with patients treated with conventional western therapy (CWT), with a decrease of 23% (RR: 0.77; 95% CI: 0.70–0.84; *P* < 0.00001) (Figure 4).

#### 3.4.2. The Complication Rate

**Figure 5:**
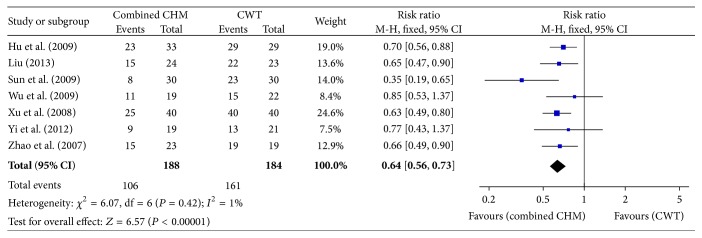
Forest plot of the complication rate.

Seven studies reported the complication rate, giving a total sample of 372 participants (188 in the combined CHM therapy group and 184 in the CWT group). The data were analyzed using a fixed-effects model according to the heterogeneity test result (*P* = 0.42, *I* ^2^ = 1%). There was significant difference between combined CHM therapy group and conventional western therapy (CWT) group with the RR of 0.64 (95% CI: 0.56–0.73; *P* < 0.00001) (Figure 5).

#### 3.4.3. Publication Bias

**Figure 6:**
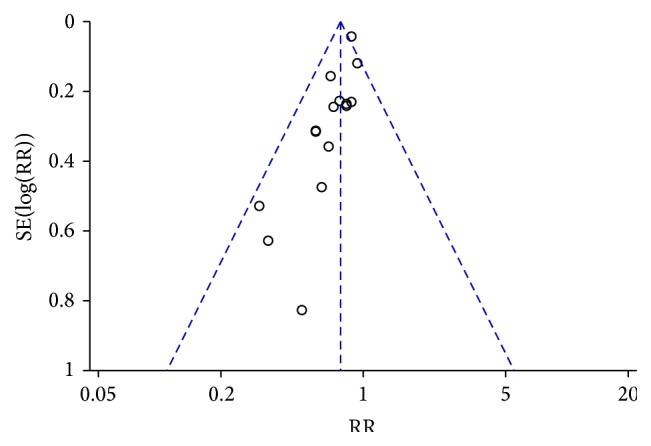
Funnel plot of all-cause mortality.

Because no sufficient number of studies reported the complication rate, we failed to perform funnel plot to detect publication bias on this outcome.  Figure 6 was the funnel plot based on studies with data on the all-cause mortality. Results showed that all points in the funnel plots were asymmetrical, indicating that publication bias may have existed in our study.

## 4. Discussions

The high mortality rate after CPR is an urgent problem to be resolved. Currently, the combined CHM therapy has been widely used in China, and a growing number of RCTs have reported that the combined CHM therapy is beneficial for patients after CPR in reducing mortality and improving prognosis. However, there was no systematic review or meta-analysis evaluating the therapeutic effect of combined CHM therapy. So, we conducted this meta-analysis.

Our systematic review of 15 RCTs included 1066 patients and showed a 23% reduction in all-cause mortality (RR: 0.77; 95% CI 0.70–0.84; *P* < 0.00001) in patients receiving combined CHM therapy compared with those assigned to conventional western therapy group. Secondly, combined CHM therapy was associated with significantly lower rates of complications (RR: 0.64; 95% CI: 0.56–0.73; *P* < 0.00001), which further confirmed the effectiveness of the combined CHM therapy in reducing the all-cause mortality. However, because of fewer studies we included in this endpoint and potential reporting bias existing, we should be careful to draw the conclusion that the reason for decreasing in mortality is that the combined CHM therapy could lower the complication rate. Further studies are needed to confirm our viewpoint and identify reasons for the reduction of mortality.

It is a very unique and complex pathophysiological process for patients after CPR, involving multiple organs. The whole-body ischemia-reperfusion injury is the mainly pathological mechanism of patients after CPR and at the same time the complications (such as MODS and SIRS) caused by it are the most important cause of high mortality. The conventional western medicine treatments are mainly concentrated in the treatment of primary disease leading to cardiac arrest and preventing those complications. However, almost all of resuscitated patients are faced with excessive consumption of physical function, as well as organs in an overloaded state, or it could be understood that there was an excessive destruction of the body's defense capabilities. This is the most critical starting point of CHM treatment—replenishing the consumption of physical function, improving defense capabilities of the whole body and resistance to disease. In traditional Chinese medicine (TCM), the main treatments are supplementing Qi, warming Yang, nourishing Yin, promoting blood circulation, and removing blood stasis.

In our meta-analysis, about 53% of studies used Shenfu injection (SFI), which has a positive effect in supplementing Qi and warming Yang. Clinical and experimental studies have shown that SFI could act on multiple organs and prevent ischemia-reperfusion injury [7], especially for myocardial dysfunction and brain injury [20, 27], which may be related to its significant effect on the improvement of energy metabolism, eliminating oxygen free radical, reducing Ca^2+^ overload blockage, suppression of the inflammatory reaction, and inhibition of apoptosis [14, 33].

Shengmai injection (about 13% used it in our included studies) has a perfect effect in benefiting Qi and nourishing Yin. Modern pharmacological studies have shown that it has a significant effect on strengthening the heart, boosting pressure, antiarrhythmia, and improving microcirculation [21, 28, 34].

Another 13% of studies reported the use of Xuebijing injection, which could promote blood circulation and remove blood stasis. It has a positive effect on suppressing inflammatory response, improving hemodynamics and tissue perfusion [30]. Wu et al. reported that it could reduce the number and the extent of MODS, which directly reduce the mortality.

In summary, clinical trials have showed that combined CHM therapy has a protective effect on multiple ischemic-reperfusion organs and tissues of the whole body. There were positive effects of jointing application of CHM for the treatment of CPR. Nevertheless, in consideration of the methodological quality of our included studies and the existence of bias, further studies are needed to provide more evidence to prove or refute our conclusion.

*Strength and Limitations*. This is the first systematic review/meta-analysis evaluating the therapeutic effect of the combined CHM therapy. Secondly, the number of participants was a relatively large sample size to clarify and confirm the issue. Thirdly, we strictly enforce the literature search, data extraction and analysis, in order to ensure the credibility of our results.

There were some limitations in our meta-analysis. Firstly, the methodological quality of the studies we included was not high. The main manifestations of the low and unclear methodological quality were as follows: for allocation sequence generation, only 26% (4/15) of the articles adequately mentioned the generation of random sequence. Allocation concealment was not described in any of the trials. Only 6% (1/15) of trials mentioned blinding and placebo-controlled method. All trials reported that there were no baseline differences, but only one study provided detailed information in tabular form. The number of patients at follow-up and the reasons for the failures at follow-up were not explicitly described in any of the trials. No studies involved intention-to-treat analysis (ITT). Therefore, the result of our meta-analysis should be cautiously considered due to low/unclear quality of evidence. There is hope that more clinical staff draw attention in these aspects and carry out more rigorously designed trials.

Secondly, all studies were conducted in China and published in Chinese, which indicated that there was publication bias. Meanwhile, the limitation of publication restricted the comprehension and application of CHM, especially for those readers and writers whose native language is not Chinese. The culture of Chinese medicine, as China's treasure, is extensive and profound. Therefore, we hope through more publication with foreign language to let more readers and writers recognize the benefits of combined CHM therapy and apply it.

## 5. Conclusions

This meta-analysis provides evidence suggesting that combined CHM therapy is associated with a decreased risk of mortality compared with conventional western therapy in patients after CPR. However, the findings should be interpreted cautiously in line with the overall methodological quality and potential biases of the included trials. More rigorously designed, randomized double-blind, placebo-controlled trials are necessary for further evaluating the effectiveness of combination CHM therapy.

## Figures and Tables

**Table 1:** The characteristics of the 15 studies.

Author and year	Patients (EG/CG)	The average age	Intervention of EG	Intervention of CG	Outcomes	Duration
Deng et al., 2012 [25]	53 (26/27)	EG: 62.54 ± 15.56 CG: 64.04 ± 13.84	Intensive care monitoring, conventional western therapy, Shenfu injection 50 mL + 5% 250 mL GLU, iv drip, QD	Intensive care monitoring, conventional western therapy	Mortality	7 d

He et al., 2013 [19]	250 (128/122)	Not reported	Intensive care monitoring, conventional western therapy, Shenfu injection 100 mL + 100 mL NS, iv drip, BID	Intensive care monitoring, conventional western therapy, 200 mL NS, iv drip, BID	Mortality	14 d

Hu, 2012 [24]	80 (42/38)	EG: 57.8 ± 12.3 CG: 57.8 ± 12.3	Intensive care monitoring, conventional western therapy, Shenfu injection 20 mL + 20 mL NS intravenous infusion (once every 30 min for 3 times), Shenfu injection 100 mL + 500 mL NS, iv drip	Intensive care monitoring, conventional western therapy	Mortality	3–7 d

Hu et al., 2009 [20]	62 (33/29)	EG: 64.3 ± 8.9 CG: 69.5 ± 7.8	Intensive care monitoring, conventional western therapy, Shenfu injection 40 mL, intravenous infusion, Shenfu 100 mL + 250 mL NS, iv drip	Intensive care monitoring, conventional western therapy, epinephrine 1 mg (once every 3-4 min), total amount less than 0.2 mg/kg	Mortality; the complication rate	6 h

Su et al., 2011 [23]	60 (31/29)	EG: 48.1 ± 13.2 CG: 48.1 ± 13.2	Intensive care monitoring, conventional western therapy, Shenfu injection 50 mL + 5% 100–250 mL GLU, iv drip, QD	Intensive care monitoring, conventional western therapy	Mortality	14 d

Liu, 2013 [27]	47 (24/23)	EG: 53.3 ± 8.2 CG: 52.8 ± 7.8	Intensive care monitoring, conventional therapy, prevention of complications, support and symptomatic treatment, Shenfu injection 100 mL, iv drip, QD	Intensive care monitoring, conventional therapy, prevention of complications, support and symptomatic treatment	Mortality; the complication rate	3–7 d

Xu et al., 2008 [22]	80 (40/40)	EG: 49.71 ± 18.82 CG: 49.71 ± 18.82	Intensive care monitoring, conventional therapy, Shenfu injection 20 mL + 5% 20 mL GLU intravenous infusion (once every 30 min for 3 times), Shenfu injection 100 mL + 450 mL NS, iv drip	Intensive care monitoring, conventional therapy	Mortality; the complication rate	Not reported

Zhang and Sun, 2012 [26]	62 (33/29)	EG: 48.2 ± 13.1 CG: 48.2 ± 13.1	Intensive care monitoring, conventional therapy, vasoactive drugs, therapeutic hypothermia, and so forth, Shenfu injection 50 mL + 5% 100–250 mL GLU, iv drip, QD	Intensive care monitoring, conventional therapy, vasoactive drugs, therapeutic hypothermia, and so forth	Mortality	14 d

Sun et al., 2009 [18]	60 (30/30)	Not reported	Intensive care monitoring, conventional therapy, Erhuang powder (18–21 g) ig, TID	Intensive care monitoring, conventional therapy, famotidine 20 mg iv drip	Mortality; the complication rate	7 d

Yi et al., 2012 [21]	40 (19/21)	EG: 70.16 ± 8.19 CG: 71.67 ± 9.64	Intensive care monitoring, routine applications of rescue measures and emergency medicine, Shengmai injection 60 mL iv	Intensive care monitoring, routine applications of rescue measures and emergency medicine	Mortality; the complication rate	Not reported

Zhao et al., 2007 [28]	42 (23/19)	EG: 63.0 ± 8.7 CG: 67.8 ± 7.1	Intensive care monitoring, conventional western therapy, Shengmai injection 50 mL, intravenous infusion, Shenfu 50 mL + 250 mL NS, iv drip	Intensive care monitoring, conventional western therapy	Mortality; the complication rate	Not reported

Liang et al., 2013 [31]	63 (33/30)	EG: 58 ± 26 CG: 61 ± 24	Intensive care monitoring, conventional therapy, ventilation, circulatory support, therapeutic hypothermia, anti-infection, supportive care, and so forth, Sini decoction, 50 mL, nasal feeding, BID	Intensive care monitoring, conventional therapy, ventilation, circulatory support, therapeutic hypothermia, Anti-infection, supportive care, and so forth	Mortality	14 d

Liang et al., 2014 [30]	86 (43/43)	EG: 58 ± 27 CG: 58 ± 27	Intensive care monitoring, conventional western therapy, treatment of the primary disease, Xuebijing injection 100 mL + 250 mL NS, iv drip, BID	Intensive care monitoring, conventional western therapy, treatment of the primary disease	Mortality	14 d

Wu et al., 2009 [29]	41 (19/22)	EG: 60.4 ± 11.1 CG: 61.3 ± 12.7	Intensive care monitoring, conventional western therapy, treatment of the primary disease, Xuebijing injection 50 mL, iv drip, BID	Intensive care monitoring, conventional western therapy, treatment of the primary disease	Mortality; the complication rate	7 d

Li and Liu, 2014 [32]	40 (20/20)	EG: 59.9 ± 60 CG: 59.9 ± 60	Intensive care monitoring, conventional western therapy, etiological treatment, Xuefuzhuyujiawei decoction, BID	Intensive care monitoring, conventional western therapy, etiological treatment	Mortality	7 d

EG = the experimental group = combined CHM therapy group; CG = the control group = conventional western therapy group.
